# Development and Initial Usability Evaluation of SSEndoSim, a Free, Web-Based Endodontic Diagnostic Simulator: Cross-Sectional Study

**DOI:** 10.2196/100407

**Published:** 2026-09-10

**Authors:** Sarang Suresh, Priya Rani, Feroze Kalhoro

**Affiliations:** 1 Department of Operative Dentistry, Liaquat University of Medical & Health Sciences, Jamshoro, Sindh, 71000, Pakistan, 92 3463361966

**Keywords:** dental education, endodontic diagnosis, gamification, mobile learning, simulation, serious game, System Usability Scale

## Abstract

SSEndoSim, a free, open access, mobile, web-based endodontic diagnostic simulation application for undergraduate dental education, demonstrated favorable usability rated on a System Usability Scale (SUS) witht a composite 100-point score (mean 75.30, SD 14.72) and high perceived educational value (PEV) on a 5-point scale (mean 4.62, SD 0.40) in a cross-sectional usability evaluation of final-year bachelor of dental surgery (BDS) students at Liaquat University of Medical and Health Sciences (LUMHS), Jamshoro, Pakistan, in April 2026, establishing usability and learner acceptability.

## Introduction

Endodontic diagnosis requires simultaneous pulpal and periapical diagnoses using American Association of Endodontists terminology [1], with treatment following the European Society of Endodontology S3-level guideline [2]. Undergraduate diagnostic accuracy is variable, and lectures and clinical exposure alone are insufficient to develop it consistently [3]. Simulation enables repeated engagement with curated cases as well as immediate feedback [4]. Free, browser-based tools may help where commercial simulation platforms are inaccessible.

We report the development of SSEndoSim, a free, web-based endodontic diagnostic simulator with automated scoring and structured feedback, and an initial evaluation of its usability and perceived educational value (PEV) among final-year bachelor of dental surgery (BDS) students ahead of a planned trial of learning effectiveness.

## Methods

### Application Development

**Figure 1.**
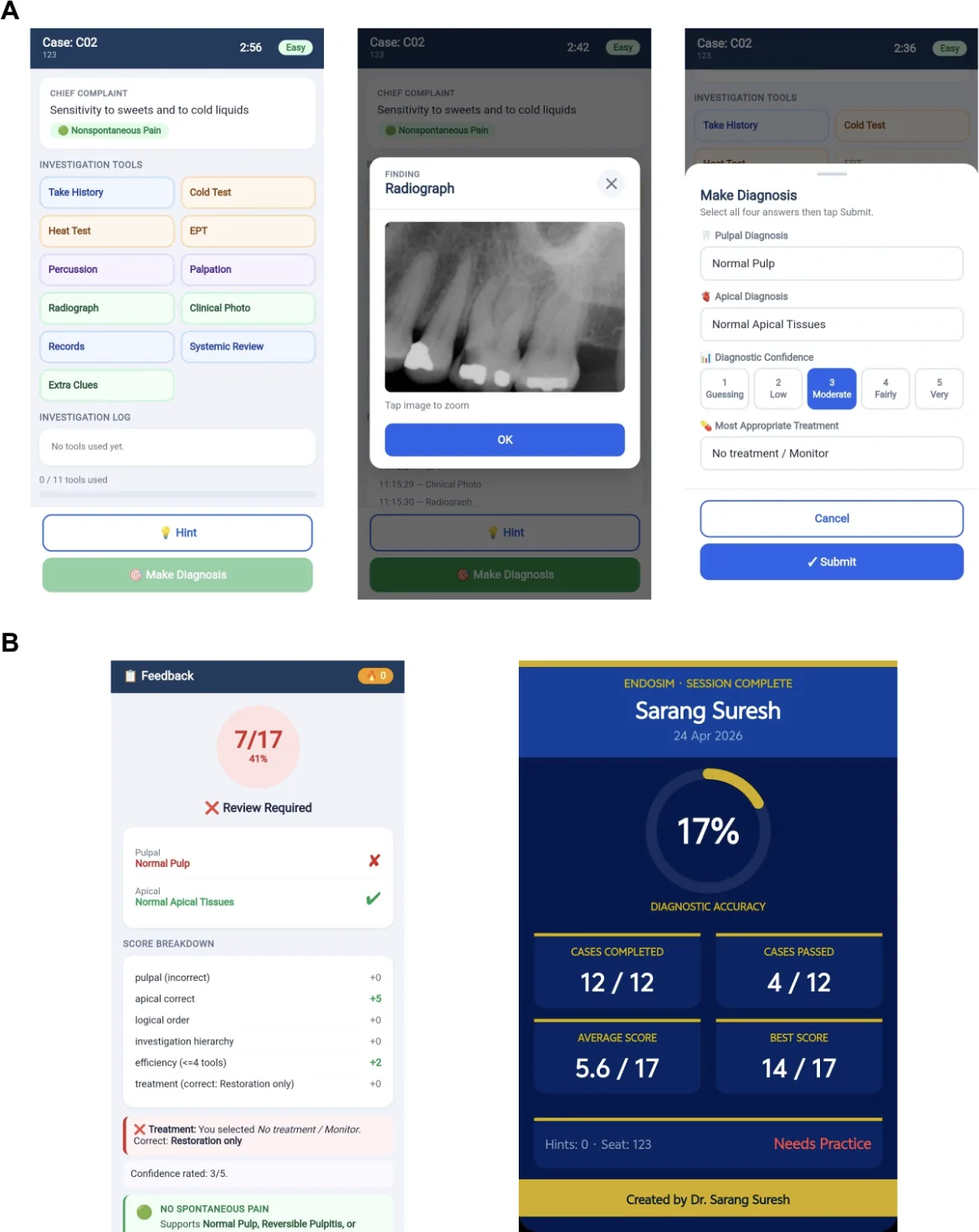
Screen captures of the SSEndoSim interface. (A) Case investigation and diagnosis workflow: investigation tool panel, radiographic finding display, and combined pulpal diagnosis, periapical diagnosis, confidence, and treatment selection panel. (B) Automated postcase feedback (diagnosis result and score breakdown) and end-of-session summary card.

SSEndoSim [5] was designed so that every case demands a simultaneous pulpal and periapical diagnosis per American Association of Endodontists terminology, and every submission receives immediate, objective feedback. It runs in any web browser without installation. The student selects investigations, submits both diagnoses, chooses treatment, and rates their confidence in the diagnoses. A 17-point rubric scores the diagnosis (10 points), treatment (2), and guideline-consistent investigation (5, with deductions), and feedback follows immediately (Figure 1). Rubric weights and difficulty labels were set by investigator judgment, and 2 senior endodontists reviewed the cases and rubric (expert review, not formal content validation). The 12-case bank spanned 6 of 7 pulpal and 4 of 6 periapical categories (4 easy, 6 moderate, and 2 hard). Cases were sampled without replacement within a session; reloading or changing difficulty began a new sequence, so cases could recur across sessions. Twenty postgraduate trainees and faculty piloted SSEndoSim informally beforehand (verbal feedback only), and their prewindow records were excluded from this study by date. SSEndoSim is for education with simulated cases, not diagnosis of real patients. Multimedia Appendix 1 details SSEndoSim’s development, rubric, case bank, and log definitions.

### Participants and Measures

All 75 students in the eligible final-year BDS cohort at Liaquat University of Medical and Health Sciences (LUMHS), Jamshoro, Pakistan, were invited to participate in this study by institutional email; those who volunteered were included. Participation was voluntary, uncompensated, and without effect on teaching, assessment, or educational opportunities. Data were collected from April 12 to 26, 2026. Usability was measured with a standard, unmodified 10-item System Usability Scale (SUS) in English [6]. PEV was measured with an exploratory 5-item investigator-developed scale (Table 1); all 15 items were scored on a scale of 1 to 5 (1=strongly disagree, 5=strongly agree). The PEV composite (participant means averaged; Cronbach *α*=.61) is exploratory; item-level results are primary. Sessions were reconstructed from log time using a 30-minute inactivity threshold, on which the session count depends. Because the questionnaire (roll number) and application (seat identifier) used different identifiers, individual SUS scores could not be linked to usage, so the SUS could not be examined by exposure level. Free-text comments were grouped into recurring topics by 1 investigator (descriptive summary; Multimedia Appendix 1). Analyses are descriptive (Python, version 3.11; Python Software Foundation), and no sample-size calculation was conducted.

**Table 1.** System Usability Scale (SUS)^a^ and perceived educational value (PEV)^b^ item results 33 final-year dental students at Liaquat University of Medical and Health Sciences (LUMHS), Jamshoro, Pakistan.

Item	Statement	Mean (SD), scale of 1 to 5
SUS1	I think that I would like to use SSEndoSim frequently.	4.61 (0.83)
SUS2	I found SSEndoSim unnecessarily complex.	1.85 (0.97)
SUS3	I thought SSEndoSim was easy to use.	4.06 (1.17)
SUS4	I think I would need technical support to use SSEndoSim.	1.91 (1.16)
SUS5	I found the various functions in SSEndoSim were well integrated.	4.00 (0.97)
SUS6	I thought there was too much inconsistency in SSEndoSim.	2.03 (1.13)
SUS7	I would imagine that most people would learn to use SSEndoSim very quickly.	4.21 (1.14)
SUS8	I found SSEndoSim very cumbersome to use.	2.21 (1.24)
SUS9	I felt very confident using SSEndoSim.	4.09 (0.98)
SUS10	I needed to learn a lot of things before I could get started with SSEndoSim.	2.85 (1.48)
PEV1	SSEndoSim helped me understand endodontic diagnostic classification better than reading alone.	4.36 (0.86)
PEV2	The immediate automated feedback after each case was educationally valuable.	4.76 (0.50)
PEV3	The scoring system motivated me to think carefully before submitting a diagnosis.	4.70 (0.64)
PEV4	I would recommend SSEndoSim to fellow dental students for self-directed learning.	4.76 (0.44)
PEV5	SSEndoSim should be included as a formal component of the endodontics curriculum.	4.52 (0.71)

a SUS composite score (0‐100): mean 75.30, SD 14.72.

b PEV composite score (1-5, exploratory): mean 4.62, SD 0.40.

### Ethical Considerations

The research ethics committee of LUMHS, Jamshoro, approved this study (LUMHS/REC/1575; March 15, 2026). Invitations were sent and subsequent written electronic consent sought through the institutional email and questionnaire system. Roll numbers, used only to screen duplicates, were deleted before analysis. Deidentified data were held by the principal investigator alone, who lectured the cohort, had no assessment role, and did not know who participated.

## Results

Of 75 invited students, 33 (44%) attempted at least 1 case and submitted a complete questionnaire; of these, 17 (52%) completed all 12 cases. Logs recorded 43 sessions and 423 scored case attempts, comprising 301 unique user-case combinations and 122 repeats, with 16 users (48%) repeating at least 1 case. The median number of unique cases attempted was 12 (IQR 5.5‐12), and the median time spent using the app was 22.4 minutes (IQR 10.7‐29.0).

The mean SUS score was 75.30 (SD 14.72; 95% CI 70.08‐80.52; median 80.0, IQR 66.2‐87.5; range 45.0‐100.0). The distribution showed no strong evidence of departure from normality (Shapiro-Wilk *P*=.35). Individual scores were as follows: 3 (9%) scored below 51, 7 (21%) score from 51 to 67, 6 (18%) scored from 68 to 79, 10 (30%) scored from 80 to 89, and 7 (21%) scored 90 or above (Multimedia Appendix 1). Item means are shown in Table 1; among the negatively worded items, the onboarding item scored highest (2.85, SD 1.48). PEV was high (mean 4.62, SD 0.40), with automated feedback and peer recommendation scoring highest (both 4.76). Of the 19 free-text comments received, the most common topics were feedback and scoring clarity (n=4; Multimedia Appendix 1).

## Discussion

SSEndoSim’s mean SUS score (75.30, SD 14.72) is comparable to other endodontic and dental diagnostic education tools evaluated with the SUS: the Endo 10 endodontic diagnosis app (median 77.5) [7], an endodontic e-learning application (81.4) [8], and a virtual scenario–based clinical-reasoning system for dental students (70.14) [9]. Samples and administration differ, so values are broadly consistent, not directly comparable. Participants rated the feedback favorably, and immediate feedback is one component associated with deliberate practice [4]; however, these data do not show that use of the app resulted in corrected diagnostic errors in users. Score-based elements may encourage use, but engagement alone does not establish learning benefit [10,11], and high recommendation and curricular-integration ratings reflect learner perception, not curriculum readiness. The open-ended data and scores for the onboarding item point to onboarding as the clearest usability target, and strengthening introductory guidance is the immediate development priority.

This single-institution study used a voluntary, self-selected sample of participants who were possibly more receptive to mobile learning than nonparticipants. Because the developer taught the cohort, perceived-authority and social-desirability effects cannot be excluded. The PEV measure was not pretested and showed modest internal consistency. Logs could not be linked to questionnaire responses, exposure varied, and only 17 participants completed all 12 cases. There was no comparator group or pre-post learning measure. The findings, therefore, concern usability and learner perception, not educational effectiveness.

A free, web-based, guideline-aligned simulator was usable and well regarded by final-year BDS students in a resource-constrained setting. Its browser-based architecture and teacher-editable case bank may be adaptable to other areas of dental education, although this was not examined here. Whether SSEndoSim improves diagnostic competence remains to be tested.

All 15 items were scored on a 5-point Likert scale (1=strongly disagree, 5=strongly agree). Values are raw item means (SD) and are not transformed for display. SUS items 2, 4, 6, 8, and 10 are negatively worded: lower raw means indicate more favorable responses, and these items are reverse scored only when computing the SUS composite. The PEV composite is the mean of each participant’s 5 PEV items, averaged across participants, and is exploratory (Cronbach *α*=.61). All values are descriptive; no hypothesis tests were performed. Full response distributions can be found in Multimedia Appendix 1.

## Supplementary material

10.2196/100407 Multimedia Appendix 1 Development account, 17-point scoring rubric, case-bank composition, application log definitions, distribution of individual SUS scores by band, response distributions for all items, and open-ended comment categories.

## Abbreviations

BDS: bachelor of dental surgery
LUMHS: Liaquat University of Medical and Health Sciences
PEV: perceived educational value
SUS: System Usability Scale
