# Supplementary material for: Development and Initial Usability Evaluation of SSEndoSim, a Free, Web-Based Endodontic Diagnostic Simulator: Cross-Sectional Study
Source: JMIR Hum Factors. 2026 Sep 10;13:e100407. doi: 10.2196/100407 (PMC13561046; doi:10.2196/100407)
Supplement: Supplementary file 1 [file humanfactors-v13-e100407-s001.docx]

**Supplementary File**

# **Development Account**

EndoSim was built by the principal investigator (SS) over approximately one year. Two design requirements were fixed at the outset. First, every case must require a simultaneous pulpal and periapical diagnosis, because the AAE consensus treats these as a paired judgment and undergraduates commonly report only one. Second, every submission must return itemized feedback without instructor involvement, so that the tool remains useful during unsupervised self-study.

The application is a single-page browser client (HTML5, CSS3, JavaScript) deployed through Google Apps Script, with cases stored in a connected Google Sheets database so that teachers can add or edit cases without redeployment. No installation, login credentials, or institutional account are required; students enter a self-chosen number identifier.

**Rubric derivation.** The 17-point allocation was set by the principal investigator to encode a teaching priority rather than an empirical weighting: the paired diagnosis carries 10 of 17 points (5 pulpal, 5 periapical) because it is the terminal judgment the rubric is designed to train; treatment selection carries 2 points as a consequence of that diagnosis; and the remaining process points reward guideline-consistent behavior. The weights were not derived from a psychometric procedure, and no formal weighting study was conducted. A psychometric evaluation of the rubric is planned alongside the effectiveness trial.

**Difficulty classification.** Difficulty was assigned by investigator judgment, principally on whether a case carried concurrent pulpal and periapical pathology and how directly the findings discriminated between candidate diagnoses. No formal criteria or external rating procedure were applied. Observed performance during the study period was ordered consistently with the assigned labels: both diagnoses correct in 59% of easy attempts (n=167), 49% of moderate attempts (n=191), and 28% of hard attempts (n=65).

**Expert review.** Two senior endodontists reviewed the case content and the scoring rubric against current AAE terminology and the ESE S3-level guideline. The review was informal: the reviewers worked jointly rather than independently, no rating instrument was used, no agreement statistic was computed, and points raised were resolved by discussion and applied directly by the principal investigator.

**Pilot.** Between April 4 and 11, 2026, 20 volunteers (postgraduate trainees and faculty members, not members of the final-year cohort) used the application informally to surface faults and gave verbal feedback. No structured protocol, task list, or written data-collection instrument was used, and no questionnaire was administered. Their records precede the study window and were excluded from the analytic dataset by date; the analytic dataset was restricted to sessions logged between April 12 and 26, 2026, and no pilot participant appears in it. Faults reported during the pilot were corrected before the study window; because feedback was verbal and informal, no change log was retained, and the specific modifications cannot be reported retrospectively.

# **Scoring Rubric (17 Points Per Case)**

| **Points** | **Criterion** | **Rationale** |
| --- | --- | --- |
| +5 | Correct pulpal diagnosis | AAE classification |
| +5 | Correct periapical diagnosis | AAE classification |
| +2 | Correct treatment selection | ESE evidence-based pathway |
| +2 | Logical investigation order (history taken first) | Pedagogical sequence criterion |
| +2 | Efficiency (four or fewer investigation tools used) | Discourages over-investigation |
| +1 | Thermal test before percussion | ESE investigation hierarchy |
| -1 | Hint accessed | Penalty for unsupported decision-making |
| -1 | Over-investigation (more than seven tools used) | Efficiency penalty |

Maximum 17 points per case; a score of 50% or above (8.5 points) is displayed as a pass. The itemized breakdown is shown to the student immediately after submission.

# **Case-Bank Composition (Study Period)**

The bank available between April 12 and 26, 2026 comprised 12 cases (C01-C12): 4 easy, 6 moderate, and 2 hard.

| **Pulpal diagnosis (AAE)** | **Cases** |
| --- | --- |
| Normal pulp | 2 |
| Reversible pulpitis | 1 |
| Symptomatic irreversible pulpitis | 4 |
| Asymptomatic irreversible pulpitis | 0 (not represented) |
| Pulp necrosis | 3 |
| Previously treated | 1 |
| Previously initiated therapy | 1 |

| **Periapical diagnosis (AAE)** | **Cases** |
| --- | --- |
| Normal apical tissues | 6 |
| Symptomatic apical periodontitis | 3 |
| Asymptomatic apical periodontitis | 0 (not represented) |
| Acute apical abscess | 2 |
| Chronic apical abscess | 1 |
| Condensing osteitis | 0 (not represented) |

Three categories were not represented because no suitable authored case had completed expert review before the study window.

# **Application Log Definitions**

A case attempt is a single case submitted for scoring; the application writes one log row at submission, so unsubmitted or abandoned cases generate no record. If the three-minute timer expires, the diagnosis panel opens and the case is still submitted and scored. A session is a continuous period of use; sessions were reconstructed from log timestamps using a 30-minute inactivity gap. Users were identified by the self-entered seat identifier. Unique cases attempted is the count of distinct case identifiers per user; repeats are attempts beyond the first on the same case.

Case selection is scoped to the in-memory session state: within an uninterrupted session, the application draws only from cases not yet seen in that session. Reloading the application, logging in again, or changing the difficulty filter resets that state and begins a new sampling sequence, so a case may recur across sessions. This behavior accounts for both the 122 repeated attempts observed and the repetition reported by participants.

| **Log measure (April 12-26, 2026)** | **Value** |
| --- | --- |
| Distinct users | 33 |
| Sessions | 43 |
| Total scored case attempts | 423 |
| Unique user-case combinations | 301 |
| Repeated attempts | 122 |
| Users with at least one repeat | 16 (48%) |
| Users completing all 12 unique cases | 17 (52%) |
| Median unique cases per user | 12 (IQR 5.5-12) |
| Median total time per user | 22.4 min (IQR 10.7-29.0) |

# **Distribution of Individual SUS Scores by Band (N=33)**

Bands follow the adjective rating scale of Bangor et al.[1] Individual SUS scores ranged from 45.0 to 100.0 (median 80.0).

| **SUS score** | **Band** | **n** | **%** |
| --- | --- | --- | --- |
| <51 | Worst imaginable / poor | 3 | 9.1 |
| 51-67 | OK / marginal | 7 | 21.2 |
| 68-79 | Good | 6 | 18.2 |
| 80-89 | Excellent | 10 | 30.3 |
| 90 or above | Best imaginable | 7 | 21.2 |

# **Response Distributions for All Items (N=33)**

Counts and percentages of participants selecting each response option for all 15 items. Response options: 1=strongly disagree, 2=disagree, 3=neutral, 4=agree, 5=strongly agree. Values are raw, untransformed responses; SUS items 2, 4, 6, 8, and 10 are negatively worded, so responses of 1 or 2 on those items are favorable.

| **Item** | **1** | **2** | **3** | **4** | **5** |
| --- | --- | --- | --- | --- | --- |
| SUS1 | 1 (3%) | 0 (0%) | 1 (3%) | 7 (21%) | 24 (73%) |
| SUS2 | 14 (42%) | 13 (39%) | 4 (12%) | 1 (3%) | 1 (3%) |
| SUS3 | 2 (6%) | 2 (6%) | 3 (9%) | 11 (33%) | 15 (45%) |
| SUS4 | 16 (48%) | 9 (27%) | 5 (15%) | 1 (3%) | 2 (6%) |
| SUS5 | 1 (3%) | 1 (3%) | 6 (18%) | 14 (42%) | 11 (33%) |
| SUS6 | 15 (45%) | 6 (18%) | 9 (27%) | 2 (6%) | 1 (3%) |
| SUS7 | 1 (3%) | 3 (9%) | 3 (9%) | 7 (21%) | 19 (58%) |
| SUS8 | 12 (36%) | 9 (27%) | 8 (24%) | 1 (3%) | 3 (9%) |
| SUS9 | 0 (0%) | 3 (9%) | 5 (15%) | 11 (33%) | 14 (42%) |
| SUS10 | 9 (27%) | 6 (18%) | 4 (12%) | 9 (27%) | 5 (15%) |
| PEV1 | 0 (0%) | 1 (3%) | 5 (15%) | 8 (24%) | 19 (58%) |
| PEV2 | 0 (0%) | 0 (0%) | 1 (3%) | 6 (18%) | 26 (79%) |
| PEV3 | 0 (0%) | 1 (3%) | 0 (0%) | 7 (21%) | 25 (76%) |
| PEV4 | 0 (0%) | 0 (0%) | 0 (0%) | 8 (24%) | 25 (76%) |
| PEV5 | 0 (0%) | 0 (0%) | 4 (12%) | 8 (24%) | 21 (64%) |

The bimodal distribution of SUS10 (9 participants strongly disagreeing and 9 agreeing) indicates that the amount to be learned before starting divided the group, consistent with the open-ended requests for introductory guidance.

# **Open-Ended Comment Categories**

Of 33 participants, 19 answered the optional question “What one feature would you add or change to improve EndoSim?” One investigator read all responses and grouped them into recurring topics after reading; categories were derived from the responses rather than defined in advance, were not mutually exclusive (one comment raised two topics), and no second coder was involved. This was a descriptive summary, not a formal qualitative analysis.

| **Topic** | **Comments** |
| --- | --- |
| Feedback or scoring clarity and detail | 4 |
| Case repetition across sessions | 2 |
| Radiographs or photographs not displaying | 2 |
| Timer or tool-access constraints | 2 |
| Introductory guidance or tutorial | 1 |
| Case history detail | 1 |
| Additional treatment options | 1 |
| Session persistence and navigation | 1 |
| No change suggested | 3 |
| Too brief to categorize | 2 |

# reference

[1]	A. Bangor, P. Kortum, and J. Miller, ‘Determining what individual SUS scores mean: adding an adjective rating scale’, *J Usability Stud.*, vol. 4, no. 3, pp. 114–123, May 2009.
